# Supplementary material for: Assessment of knowledge and perceptions towards diabetes mellitus and its associated factors among people in Debre Berhan town, northeast Ethiopia
Source: PLoS One. 2020 Oct 19;15(10):e0240850. doi: 10.1371/journal.pone.0240850 (PMC7571671; doi:10.1371/journal.pone.0240850)
Supplement: S1 File — (DOCX) [file pone.0240850.s001.docx]

S1 File. English Version Questionnaire

Questionnaire ID No.__________

Instruction: Please circle the number in front of the option the respondents choose & fill in the blank space that the respondent best describe in the right side of the table.

| **Part-I: Respondent’s socio- demographic information** | | | |
| --- | --- | --- | --- |
| S.No | Questions | Response options | Remarks |
| 100 | Sex | 0 = Male  1 = Female |  |
| 101 | Age | ________Years old |  |
| 102 | Marital status | 0 = Single  1 = Married  2 = Divorced/Separated  3 = Widowed |  |
| 103 | Level of education | 0 = Unable to read and write  1 = Grade 1-4  2 = Grade 5-8  3 = Grade 9-12  4 = College and above |  |
| 104 | Occupation | 0= House wife  1 = Husband  2= Student  3 = Merchant  4 = Farmer  5 = Government/private employee  6= Daily laborer  7= Other(specify)_____________ |  |
| 105 | Average family monthly income | _______________(in Ethiopian birr) |  |
| 106 | Exposure to health education about DM | 0 = Yes  1 = No |  |
| 107 | If “Yes” for Q-106 your sources of information? | 0 = Medias  1 = Health care workers  2 = Friends/relatives  3 = Others (teacher, religious leader…) |  |
| 108 | Family history of DM | 0 = Yes 1 = No 2 = Do+`n’t know |  |

| **Part-II: Knowledge questions related to DM** | | | | | |
| --- | --- | --- | --- | --- | --- |
| S.No | Questions | Response options | | | |
|  |  | Yes =1 | No= 2 | I don’t know = 3 | Remark |
|  | **What is/are DM** |  |  |  |  |
| 200 | DM is a condition of insufficient insulin production |  |  |  |  |
| 201 | DM is a condition of the body which not responding for insulin |  |  |  |  |
| 202 | DM is a condition of high level of sugar in the blood |  |  |  |  |
| 203 | DM is not curable |  |  |  |  |
|  | **What are the risk factors of DM** |  |  |  |  |
| 204 | Older age |  |  |  |  |
| 205 | Genetic or family history of diabetes mellitus |  |  |  |  |
| 206 | Being overweight /Obesity |  |  |  |  |
| 207 | Sedentary life /Poor dietary habits |  |  |  |  |
|  | **What are the signs and symptoms of DM** |  |  |  |  |
| 208 | Frequent urination |  |  |  |  |
| 209 | Excessive thirst |  |  |  |  |
| 210 | Excessive hunger |  |  |  |  |
| 211 | Weight loss |  |  |  |  |
| 212 | High blood sugar |  |  |  |  |
| 213 | Blurred vision |  |  |  |  |
| 214 | Slow healing of cuts and wounds |  |  |  |  |
| 215 | Feeling of weakness |  |  |  |  |
|  | **Control and management of DM** |  |  |  |  |
| 216 | Insulin injection is available for control and management of DM |  |  |  |  |
| 217 | Tablets & capsule are available for control and management of DM |  |  |  |  |
| 218 | Regular Exercise |  |  |  |  |
| 219 | Practices healthy diet |  |  |  |  |
| 220 | Weight reduction |  |  |  |  |

**Part III. Questions on Perception about diabetes mellitus based on HBM**

|  | **Questions** | Response scale | | | | |
| --- | --- | --- | --- | --- | --- | --- |
|  | **perceived susceptibility** | Strongly agree(SA) | Agree (A) | Uncertain (U) | Disagree (D) | Strongly disagree(SD) |
| 300 | My chances of getting diabetes in next few years is great |  |  |  |  |  |
| 301 | I feel I will get diabetes sometime during my life |  |  |  |  |  |
| 302 | I believe all population are equally likely to develop diabetes |  |  |  |  |  |
|  | **Perceived severity** |  |  |  |  |  |
| 303 | If I had diabetes , I would be worried and depressed |  |  |  |  |  |
| 304 | If I had diabetes , I would have to have my diabetes taken off by anti-diabetes medication |  |  |  |  |  |
| 305 | Diabetics can be a serious disease if you don’t prevent it. |  |  |  |  |  |
| 306 | If I had diabetes, it would cause me to die |  |  |  |  |  |
|  | **Perceived Benefits** |  |  |  |  |  |
| 307 | I believe diabetes can be cured easily |  |  |  |  |  |
| 308 | I believe maintain a normal body weight help to control diabetes |  |  |  |  |  |
| 309 | Regular health care visit will help finding diabetes early and can help save my life |  |  |  |  |  |
| 310 | I believe that eat low sugar snacks & low-fat meals prevent diabetes in the future |  |  |  |  |  |
| 311 | I believe that regularly physical exercise will help to prevent diabetes |  |  |  |  |  |
|  | **Perceived Barriers to screening and healthy lifestyle** |  |  |  |  |  |
| 312 | I don't want to know if I have diabetes or not. |  |  |  |  |  |
| 313 | I think having a regular health check-up takes too much time. |  |  |  |  |  |
| 314 | Not having enough money would keep me from having a check-up. |  |  |  |  |  |
| 315 | I never heard or read anything encouraging having regular health check-up. |  |  |  |  |  |
| 316 | If I find I have diabetes, people will treat me differently, so I don't want to have diagnosis. |  |  |  |  |  |
| 317 | I could not have enough of time to exercise |  |  |  |  |  |

We have finished thank you very much!
