# Supplementary material for: Assessment of knowledge and perceptions towards diabetes mellitus and its associated factors among people in Debre Berhan town, northeast Ethiopia
Source: PLoS One. 2020 Oct 19;15(10):e0240850. doi: 10.1371/journal.pone.0240850 (PMC7571671; doi:10.1371/journal.pone.0240850)
Supplement: S2 File — (DOCX) [file pone.0240850.s002.docx]

S2 File. Amharic Version Questionnaires

**ቃለ መጠይቅ**

የመረጃ ሰብሳቢው አደራሻ:

የመልስ ሰጪዉ ፊርማ________________ቀን _____________________

መጠይቅ የተጀመረበት ቀን : ________ሰዓት: _______ያለቀበት ሰዓት: _________.

የሱፐርቫይዘሩ ስም ________________________ፊርማ _________

ትዕዛዝ: እባክዎ መላሹ የሚሰጠዉን መልስ ከጥያቄዎች ፊት ለፊት ካሉት አማራጮቹ መካከል ያክብቡ ወይም መላሹ የሚገልፀዉን ሀሳብ በቀኝ በኩል ባለዉ ክፍት ቦታ ላይ ይፃፉ፡፡

| ክፍል-አንድ: የጥናቱ ተሳታፊዎች ማህበራዊ ኩነታት መረጃዎች | | | | | | | | |  |  |
| --- | --- | --- | --- | --- | --- | --- | --- | --- | --- | --- |
| ተ.ቁ | | ጥያቄ | የመልስ አማራጮች | | | | ምርመራ | |  |  |
| 100 | | ጾታ | 0 = ወንድ  1 = ሴት | | | |  | |  |  |
| 101 | | ዕድሜ | ________ዓመት | | | |  | |  |  |
| 102 | | የጋብቻ ሁኔታ | 0 = ያላገባ  1 = ያገባ  2 = የፈታ/የፈታች/ለየብቻ የምኖሩ  3 = የሞተባት/የሞተችበት | | | |  | |  |  |
| 103 | | የትምህርት ደረጃ | 0 = ማንበብናመፃፍ የማይችል  1 = ክፍል 1-4  2 = ክፍል 5-8  3 = ክፍል 9-12  4 = ኮሌጅ እና ከዚያ በላይ | | | |  | |  |  |
| 104 | | የስራ ሁኔታ | 0= የቤት እመቤት  1 = ተማሪ  2 = ነጋዴ  3 = አርሶ አደር/አርብቶ አደር  4 = የመንግስት/የግል ተቀጣሪ  5 = የቀን ሰራተኛ  6 = ሌላ(ይገለፅ)_____________ | | | |  | |  |  |
| 105 | | አማካይ የቤተሰብ ወርሃዊ ገቢ | _______________ብር ( ኢትዮጵያ ብር) | | | |  | |  |  |
| 106 | | ስለስኳር በሽታ የጤና ትምህርት አገኚተዉ ያዉቃሉ | 0 = አዎ  1 = የለም | | | |  | |  |  |
| 107 | | ጥያቄ ቁጥር -106 መልስዎ “አዎ” ከሆነ መረጃዉን ያገኙት ከየት ነዉ? | 0 = መገናኛ ብዙሃን  1 = የጤና ባለሞያ  2 = ጓደኛ/ዘመድ  3 = ሌላ (መምህር, የሀይማኖት አባት…) | | | |  | |  |  |
| 108 | | በቤተሰብ ዉስጥ የስኳር በሽታ የተያዘ አለ | 0 = አዎ  1 = የለም  2 = አላዉቅም | | | |  | |  |  |
| **ክፍል-ሁለት: የስኳር በሽታ እዉቀትን የሚመለከቱ ጥያቄዎች** | | | | | | | | | | |
| ተ.ቁ | | ጥያቄዎች | | | የመልስ አማራጮች | | | | | |
|  |  |  |  |  | አዎ =1 | አይደለም  =2 | አርግጠኛ አደለሁም  = 3 | | ምርመራ | |
|  | | **የስኳር በሽታ ማለት ምን ማለት ነው ?** | | |  |  |  | |  | |
| 200 | | የስኳር በሽታ ማለት ከቆሽት የሚመነጨው ኢንሱሊን የተባለው ሆርሞን (ንጥረ ነገር) ጭራሽ መጥፋቱ/አለመመንጨቱ ወይም መጠኑ መቀነሱ | | |  |  |  | |  | |
| 201 | | የስኳር በሽታ ማለት ከቆሽት የሚመነጨው ኢንሱሊን የተባለው ሆርሞን (ንጥረ ነገር) የሚያከናውነው ሥራ ሲሰናከል የሚመጣ ነዉ። | | |  |  |  | |  | |
| 202 | | የስኳር በሽታ ማለት ስኳር ወይም ጉሉኮስ በደም ውስጥ ከመጠን በላይ ሆኖ ሲገኝ የሚከሰት ነው፡፡ | | |  |  |  | |  | |
| 203 | | የስኳር በሽታ መዳን የማይችል ነዉ፡፡ | | |  |  |  | |  | |
|  | | **ለስኳር በሽታ መከሰት ምክንያት ሊሆኑ የሚችሉ ጠንቆች** | | |  |  |  | |  | |
| 204 | | የዕድሜ መጨመር (በእድሜ መግፋት) | | |  |  |  | |  | |
| 205 | | ዘር ወይም በቤተሰብ ውስጥ ሕመሙ ቀደም ብሎ መታየቱ | | |  |  |  | |  | |
| 206 | | ዉፍረት(ውፋሬ) ወይም ክብደት ከሚገባ በላይ መጨመር | | |  |  |  | |  | |
| 207 | | የልተስተካከል የኑሮ ሁኔታ/የአመጋገብ ሁኔታ | | |  |  |  | |  | |
|  | | **የስኳር በሽታ ምልክቶች ምን ምን ናቸው?** | | |  |  |  | |  | |
| 208 | | አዘውትሮ /ቶሎ ቶሎ መሽናት | | |  |  |  | |  | |
| 209 | | በብዛት ውሃ መጠጣት | | |  |  |  | |  | |
| 210 | | በብዛት መራብ | | |  |  |  | |  | |
| 211 | | በብዛት ክብደት መቀነስ (መክሳት) | | |  |  |  | |  | |
| 212 | | በደም ዉስጥ ያለዉ ስኳር መጠን በብዛት መጨመር | | |  |  |  | |  | |
| 213 | | የዓይን ግርዶሽ | | |  |  |  | |  | |
| 214 | | በሰዉነት ላይ ያሉ ቁስሎች ቶሎ አለመዳን/ከቁስል በቶሎ ለመዳን አለመቻል | | |  |  |  | |  | |
| 215 | | የድካም ስሜት | | |  |  |  | |  | |
|  | | **የስኳር በሽታንና ጠንቆቹን እንዴት መከላከል ወይም እንዳይባባሱ ማድረግ ይቻላል?** | | |  |  |  | |  | |
| 216 | | በመርፌ መልክ በሚወሰዱ የተለያዩ የኢንሱሊን ዝግጅቶች የስኳር በሽታን መቆጣጠር ይቻላል | | |  |  |  | |  | |
| 217 | | በአፍ በሚወሰዱ በክኒን እና በጥቅል መልክ ባሉ መድሀኒቶች የስኳር በሽታን መቆጣጠር ይቻላል | | |  |  |  | |  | |
| 218 | | አዘውትሮ የሰውነት እንቅስቃሴ (ስፖርት) መሥራት። ለምሳሌ ያህል በየቀኑ ግማሽ ስዓት በመውሰድ በሳምንት ውስጥ ለአምስት ቀናት ያህል መሮጥ | | |  |  |  | |  | |
| 219 | | ጤነኛ የሆነ ምግብ መመገብ(ይህ ምግብ አትክልትና ፍራፍሬ በብዛት የያዘ) | | |  |  |  | |  | |
| 220 | | ተገቢ በሆነ የሰውነት ክብደት ወይም ውፋሬ ላይ መገኘት | | |  |  |  | |  | |

**ክፍል 3: በጤና እምነት ሞዴል ላይ የተመሰረተ ግንዛቤን በተመለከተ የተዘጋጀ መጠይቅ**

|  | ጥያቄዎች | የምላሽ ልኬት መለኪያ | | | | |
| --- | --- | --- | --- | --- | --- | --- |
|  | **የተጋላጭነት** ስሜት **ተገንዝቧል** | በጣም እስማማለሁ | እስማማለሁ | ገለልተኛ | አልስማማም | በጣም አልስማማም |
| 300 | በቀጣዮቹ ጥቂት አመታት ውስጥ በስኳር በሽታ የመያዝ እድሌ በጣም ከፍተኛ ነው፡፡ |  |  |  |  |  |
| 301 | በህወት ዘመኔ የስኳር በሽታ አንድ ጊዜ እንደሚይዘኝ አሰባለሁ፡፡ |  |  |  |  |  |
| 302 | ሁሉም ማህበረሰብ በስኳር በሽታ የመያዝ እድሉ እኩል እንደሆነ አምናለሁ፡፡ |  |  |  |  |  |
|  | **የደረሰብህ/ሽ ጉዳት** |  |  |  |  |  |
| 303 | የስኳር በሽታ ቢኖርብኝ እጨነቅና እሰጋ ነበር፡፡ |  |  |  |  |  |
| 304 | የስኳር ህመም ቢኖርብኝ መድሃኒት መውሰድ እንዳለብኝ አስባለሁ፡፡ |  |  |  |  |  |
| 305 | የስኳር ህመም ከባድ በሽታ በመሆኑ መከላከል አይቻልም፡፡ |  |  |  |  |  |
| 306 | የስኳር በሽታ ካለብኝ እሞታለሁ፡፡ |  |  |  |  |  |
|  | **የሚገኙ ጥቅሞች** |  |  |  |  |  |
| 307 | የስኳር በሽታን በቀላሉ መዳን እንደሚቻል አምናለሁ፡፡ |  |  |  |  |  |
| 308 | የስኳር በሽታን ለመቆጣጠር መደበኛ የሰውነት ክብደት አስፈላጊ እንደሆነ አምናለሁ፡፡ |  |  |  |  |  |
| 309 | መደበኛ የጤና ክትትል ማድረግ ቀድሞ የስኳር በሽታ መኖሩን ለመረዳትና ህወቴን ለማዳን ይረዳኛል፡፡ |  |  |  |  |  |
| 310 | ዝቅተኛ የቅባትና የስኳር መጠን ያላቸው ምግቦችን በመመገብ ወደፊት የስኳር በሽታን መከላከል እንደሚቻል አምናለሁ፡፡ |  |  |  |  |  |
| 311 | መደበኛ አካላዊ እንቅስቃሴ በማድረግ የሰኳር በሽታን መከላከል እንደሚቻል አምናለሁ፡፡ |  |  |  |  |  |
|  | **የጤና ምርመራ ክትትል እንዳይደረግ የሚያደርጉ ግንዛቤዎች::** |  |  |  |  |  |
| 312 | የስኳር በሽታ እንዳለብኝ ወይም እንደሌለብኝ ማወቅ አልፈልግም፡፡ |  |  |  |  |  |
| 313 | መደበኛ የሆነ የስኳር ምርመራ ማድረግ ግዜ ይወስዳል ብዬ አስባለሁ፡፡ |  |  |  |  |  |
| 314 | በቂ የሆነ ገንዘብ ስለሌለኝ መደበኛ የሆነ የስኳር ምርመራ እንዳላደርግ አድርጎኛል፡፡ |  |  |  |  |  |
| 315 | በየግዜው የጤና ምርመራ እንዳላደርግ መረጃ የለኝም ወይም አልሰማሁም፡፡ |  |  |  |  |  |
| 316 | የስኳር በሽታ ካለብኝ ሰዎች በተለዬ መንገድ ስለሚረዱኝ ምርመራ ማድረግ አልፈልግም፡፡ |  |  |  |  |  |
| 317 | አካላዊ እንቅስቃሴ ለማድረግ በቂ ግዜ የለኝም፡፡ |  |  |  |  |  |

ጨርሰናል! በጣም አመሰግናልሁ!!!!
