# Supplementary material for: Assessment of knowledge and perceptions towards diabetes mellitus and its associated factors among people in Debre Berhan town, northeast Ethiopia
Source: PLoS One. 2020 Oct 19;15(10):e0240850. doi: 10.1371/journal.pone.0240850 (PMC7571671; doi:10.1371/journal.pone.0240850)
Supplement: Attachment — Submitted filename: author response R2.docx [file pone.0240850.s004.docx]

**Author’s response to editor and reviewers**

**Title**: Assessment of Knowledge and Perception towards Diabetes Mellitus and Its Associated Factors among People in Debre Berhan Town, Northeast Ethiopia.

**Version 2**: September 30, 2020

**Dear editor**

To PLOS ONE, we are pleased to resubmit for publication version of “**Assessment of Knowledge and Perception towards Diabetes Mellitus and Its Associated Factors among People in Debre Berhan Town, Northeast Ethiopia”** with manuscript ID of “PONE-D-20-04491**”** for a review as original research in PLOS ONE. The comment of the editor was highly insightful and enabled us to greatly improve the quality of our manuscript. Therefore, based on the editor concerns we have made extensive edition in our manuscript. The formatting of the text and document (text sizes and grammatical errors) were also edited. In the following pages, we have addressed yours’ concerns in a point by point format.

We look forward to hearing from you at your earliest convenience.

Once again, thank you for considering our manuscript in PLOS ONE!

Kind regards,

**Wondimeneh Shibabaw Shiferaw**

On behalf of all the authors

**Author’s response to Editor Comments**

The manuscript has addressed the comments provided and the quality has significantly improved. However, a number of issue remain

1. The manuscript has a lot of grammatical errors and typos that need to be a addressed. Thorough copyediting by native speaker is required. Some example of error observed.
a. Abstract: Result: diabetes is a seriousness disease. Should read diabetes is a serious disease

**Response:** We thanks and accept your suggestion.

b. Abstract: results: agreed in the perceived benefits should read agreed to the perceived benefits

**Response**: Accept your suggestion

c. Abstract: Conclusion: behavior change has importance in prevention….should read behavior change is important. ..

**Response**: We accept your suggestion

d. Diabetic mellitus should ready diabetes mellitus

**Response**: We accept your suggestion

e. Results: sociodemographic: ample should read sample

**Response**: We thanks and accept your suggestion.

f. Thorough copyedit document

**Response**: Based on your suggestion, we have made extensive edition in the entire document by native speaker (Dr. Ryan Bell).

2. All references need to be properly checked and ensure the names and initials are accurate. They are several errors in the initials. Make sure the referencing is consistent with the journal requirement. Remove CAPS in the references (#33 and 42). Ensure first letter in names are appropriately capitalized. Include year in all the listed publications (#39). Correct all errors in the references esp # 2,3,4,9,13,15,17,18,19,20,23,26,30,33,42,37,39.

**Response**: Really the comment is constructive. Based on your feedback appropriate modification has been made in the entire references section in the revised manuscript.
